# Exploring the Process of Spiritual Health of the Elderly Living in Nursing Homes: A Grounded Theory Study

**DOI:** 10.4314/ejhs.v31i3.16

**Published:** 2021-05

**Authors:** Ali Jadidi, Masoud Khodaveisi, Efat Sadeghian, Masoud Fallahi-Khoshknab

**Affiliations:** 1 School of Nursing and Midwifery, Hamadan University of Medical Sciences, Hamadan, Iran; 2 Chronic Disease (Home Care) Research Center, Community Health Nursing Department, School of Nursing and Midwifery, Hamadan University of Medical Sciences, Hamadan, Iran; 3 Chronic Disease (Home Care) Research Center, Nursing Department, School of Nursing and Midwifery, Hamadan University of Medical Sciences, Hamadan, Iran; 4 Department of Nursing, University of Social Welfare and Rehabilitation Sciences, Tehran, Iran

**Keywords:** Nursing Homes, Elderly, Spirituality, Grounded Theory

## Abstract

**Background:**

Spiritual health is one of the important dimensions of the elderly's health, which plays an important role in other dimensions of their health. This study aimed to explain the process of spiritual health of the elderly living in nursing homes.

**Methods:**

This grounded theory study was conducted in 4 nursing homes in the city of Arak Iran between October 2019 and September 2020. The participants were 24 elderly people living in nursing homes, two health care providers, one nurse and one family member, first selected through purposive sampling and then, through theoretical sampling. The data were collected through semi-structured interviews and field notes. All the interviews were transcribed verbatim and analyzed based on Strauss and Corbin approach (2008).

**Results:**

Six main categories were identified, including helplessness, inefficient supportive environment, spiritual distress, seeking support, relative improvement of spiritual health and factors affecting spiritual health, each of which explains a part of the whole process of spiritual health of the elderly living in nursing homes.

**Conclusion:**

Supporting the elderly living in nursing homes is necessary in order to meet their spiritual needs and preserve and promote their spiritual health.

## Introduction

In the societies where the number of elderly people is increasing and they are not capable of taking care of themselves, transferring the elderly to nursing homes is an appropriate action. As the elderly living in nursing homes are deprived of family support, preserving and promoting their health is of great importance (1–2). One of the important dimensions of the elderly's health is spiritual health, serious attention to which is believed to be necessary by some experts (3).

Spiritual health refers to that aspect of the existence of an individual which is in search of meaning and purpose of life.

Spiritual health is a hidden concept of supernatural nature and generally encompasses perfection, ethics and disciplines, purposeful life, commitment to something superior and belief in subjective concepts (4–5). Some studies indicate that without spiritual health, other biologic, mental and social dimensions of elderly people cannot act properly or reach their maximum capacity (5).

Evidence show that as the elderly confront the disadvantages of the old age, they turn to religion and spirituality more than others (6). It has been previously shown that religion and spirituality play an important role in directing lives of the elderly, understanding meaning of life and adaptation to old age's disadvantages and the elderly consider old age as a period of spiritual growth and development (7). However, the available evidences indicate that spiritual health of the elderly living in nursing homes is lower than that of other elderly people (8). The previous studies also showed that spiritual health of the elderly living in nursing homes gradually decreases, so that their spiritual health is not at a desired level compared to other elderly people. This can be due to the lack of attention to their spiritual needs, having inappropriate understanding of spiritual health, existing limitations and insufficient knowledge of spiritual health (9–11).

Of course, it is assumed that as the elderly are adapted to living in nursing homes over time, their spiritual health improves as well. It is believed that newcomer elderly people feel lonely more and welcome group activities and programs less than others, but the elderly who have lived in nursing homes for a longer period of time are more sociable and joyful (12). Similarly, Kane et al. indicates that the elderly who have lived in nursing homes for a longer period of time are more interested in religious and spiritual programs (13). In contrast, Herlina et al. believes that spiritual health of the elderly gradually decreases and is affected by reduced physical and mental performance (14). Also, it has been demonstrated that spiritual health of the elderly living in nursing homes is low and is, most importantly, affected by their personality, religion and attitude toward life (15).

Although spiritual health is considered as one of the important dimensions of health in the elderly, it has received less attention compared to other dimensions of health in these people. Spiritual health of the elderly is a social process, of which there is limited knowledge and no comprehensive understanding to make its adequate description possible. Therefore, due to the lack of our knowledge of the process of spiritual health of the elderly living in nursing homes, this study was conducted aiming to explain the process of spiritual health of the elderly living in nursing homes using grounded theory. The main question of this study is “how is the process of spiritual health of the elderly living in nursing homes?” The findings of this study can be useful for health care providers at nursing homes, creating a new insight and increasing basic knowledge of the process of spiritual health in the elderly living in nursing homes.

## Methods

**Design**: The grounded theory is a qualitative research methodology used to investigate the social processes involved in human interactions and the structure and process leading to them (16). The Strauss and Corbin's version systematically applies specific steps and eventually achieves the theory behind the data or theoretically explains a particular phenomenon (17–18).

This qualitative study was conducted between October 2019 and September 2020 using the naturalistic paradigm through inductive reasoning and grounded theory based on Strauss & Corbin (2008).

**Participants**: The participants were selected from the elderly living in 4 nursing homes in the city of Arak, Iran. To better understand the process of spiritual health, the participants were sought to represent a wide range of individuals in terms of age, duration of residence in nursing home, education and religious commitment. Inclusion criteria were being between 60 and 90 years of age, permanent residence in nursing home, having the ability to talk and listen, having the ability to answer the interview's question in Persian, having no acute or chronic mental disease and having no serious physical illnesses.

In this study, sampling was started with purposive sampling and then, theoretical sampling was employed for collecting the data during the process of analysis. As the research progressed and initial categories emerged, the data directed the researcher to other individuals with maximum information required for completing the basic theory (including health care providers, nurse and family member). Totally, 24 elderly people, 2 health care providers, 1 nurse and 1 family member were interviewed.

**Data collection:** The main data collection method in this study included individual deep semi-structured interviews and field notes. A collection of preplanned questions related to the study's purpose, such as the following ones, were used for directing the interview process and data collection: “Describe one day of your life in nursing home, please”; “How do you communicate with others?”; “How do you communicate with God?

The interviews were performed individually and in a quiet place in an appropriate time where the participants felt at ease. Each interview lasted for 35–65 minutes and they were repeated if required (for the Participants No. 2, 9, 12 and 14). After the interviews, they were carefully listened and typed word for word as soon as possible in order to maintain the connection with the data and feelings of the participants.

**Data analysis**: The data analysis was performed at the same time as data collection. According to Strauss and Corbin (2008), the data analysis was conducted considering 4 stages: data analysis for concepts, data analysis for context, process analysis in data analysis, and integration of categories.

The stage of data analysis for concepts began with open coding. Through continuous comparisons, the extracted initial codes were categorized according to their similarities and differences. Subsequently, the emerged categories were subdivided into more abstract ones according to their characteristics and dimensions. At the same time, data analysis for context was also performed. At this stage, the influential moods and conditions were taken into consideration. In other words, the researchers were looking for the conditions leading to problems. The next analytical task was to discover the process by which individuals respond to problems with action/reaction and emotions. At this point, the researchers delved into the categories to discover the strategies and behaviors which the participants showed and used the comparative methods. The last stage was the integration of categories. The integration of categories was accomplished by writing the story line, drawing diagrams and reviewingthe reminders and the central category was finally identified. These four stages of the analysis were not linear and often occurred simultaneously.

MAXQDA 10 was used for managing and organizing the data. To analyze and identify the data representing the context, process and outcome, analytical tools such as asking questions from data (critical questions, theoretical questions, questions with operational nature and biased questions), comparing (continuous comparison and theoretical comparison), thinking of different meanings of a word and paying attention to body language and expressed feelings were used. Further, reminders were also used in the data analysis.

**Rigor**: To validate the data, the criteria proposed by Lincoln and Guba were used (19). The validity and rigor of the data were insured by reviewing the entries by the participants, integrating the data resources and method integration, confirming the coding by the colleagues familiar with qualitative research and using the colleagues' comments. In addition, the researchers carefully registered the research documentations to allow an external reviewer to evaluate the study. Also, the researchers tried to extract the common voice of the population under study using mentioning multiple quotations in the Conclusion section and provide a clear picture of the process of spiritual health of the elderly.

**Ethical considerations**: This study was approved by Research Ethics Committee of Hamedan University of Medical Sciences (IR.UMSHA.REC.1398.873). The study's objectives and methods were completely explained to the participants. Also, they were completely assured of the confidentiality of the data and their freedom to participate in the study or to leave it at any time. Time and place of the interviews were determined by the participants' agreement and based on their preferences.

## Results

Totally, 24 elderly people (11 male and 13 female) of 64–84 years of age (mean: 75.4±5.43), 2 health care providers (34 and 38 years old), 1 nurse (52 years old) and 1 member of the family of an elderly participant (45 years old) participated in this study. Average duration of residence of the elderly people in nursing homes was 5.2±1.21 years.

The analysis of the collected data in this research led to extraction of 6 main categories, including helplessness, inefficient supportive environment, spiritual distress, seeking support, relative improvement of spiritual health and factors effecting spiritual health, and 17 subcategories, each of which explains a part of the whole process of spiritual health of the elderly living in nursing homes. Data analysis and reduction was achieved during the axial coding according to the similarities and differences among the categories and also considering model paradigm (Table 1).

**Table 1:** Process of emergence of categories, subcategories and initial categories

Categories	Subcategories	Open Codes
**Helplessness**	Feeling of being abandoned	Being abounded by family, being abounded by society
	Feeling of loneliness	Having no supporter, loneliness
**Inefficient** **supportive** **environment**	Insufficient spiritual care	Limited religious practice, insufficient support
	Insufficient knowledge of health care providers	Insufficient communication knowledge, insufficient care knowledge
**Spiritual distress**	Decreased religious commitment	Less attention to praying and saying prayers, reduced belief in religious values, reduced belief in afterlife
	Disturbed understanding of meaning and purpose	Feeling of being aimless, confusion, lack of pleasure in life, ennui, suffering from living in nursing home
	Disappointment	Waiting for death, discouragement, wishing death, having no hope in life
	Lack of internal peace	Unorganized thinking, not being able to concentrate, feeling of fear and anxiety, sleep disorder
	Disturbed communication	Disturbed communication with God, disturbed communication with family members, insufficient communication with other elderly people, disturbed communication with health care providers
**Seeking support**	Reliance on supportive resources	Turning to religious resources, turning to rehabilitation resources
	Emergence of spiritual needs	Need for meaning, need for hope, need for peace
**Relative** **improvement of** **spiritual health**	Understanding spiritual pleasure	Achieving peace, hope and meaning
	Spiritual growth	Turning to religious practice, feeling God's support, strengthening beliefs
	Constructive relationship with others	Relationship with family members, relationship with elderly people, communication with health care providers
**Factors affecting** **spiritual health**	Personal factors	Spiritual background of the elderly individual, spiritual attitude of the elderly individual
	Family factors	Spiritual background of family, extent of family relationships
	Care factors	Spiritual care, spiritual attitude of health care providers

**Helplessness**: Most of the newcomer elderly in nursing homes feel frustrated as they see themselves discarded. This category includes two subcategories of abandonment and loneliness.

Many of the elderly in their interviews referred to this issue that retirement, reduced physical and mental abilities and changes in cognitive processes have led to their rejection by family and society and the others respect them less than before. For example, one of the participants mentioned that: “*When you are old, your income and thinking ability are not like before, no one cares about you any longer*” (Participant No. 9). Another participant mentioned that: “*They have taken us here [nursing home] as if we were a useless and out-of-date thing*” (Participant No. 2).

Another problem to which all the elderly referred was loneliness. Most of them were unhappy of having no wife or children and considered having no caregiver as one of the reasons for their residence in nursing home. One of the participants mentioned that: “*When my wife died, I became very alone; I had neither a child nor somebody to take care of me. I was made to come to nursing home*” (Participant No. 11).

**Inefficient supportive environment**: As mentioned by many elderly participants, nursing home is not capable of taking appropriate care of the elderly and it is not an appropriate environment in which they can feel calm and at ease. Continuing the data analysis, two subcategories of insufficient spiritual health and insufficient knowledge of health care providers were put under this main category.

Most of the elderly referred in their interviews to this issue that their spiritual needs are not appropriately met and spiritual dimension is ignored in the care provided to them. One of the participants mentioned that: “*Here, there is neither a person to help us nor a person with whom you talk, nor a place to go to feel calm and relaxed*” (Participant No. 21). Also, one of the health care providers mentioned that: “*Here, the number of the elderly people is very high, but the number of health care providers is low. We cannot take care of them better than this*” (Participant No. 15).

Also, most of the elderly complained of insufficient care and communication knowledge of health care providers and considered them unqualified in this regard. One of the participants mentioned that: “*I don't know where they have found these health care providers! They don't know how to talk properly and do nothing; they only sit and play with their mobile phones*” (Participant No. 3). Also, one nurse mentioned that: “*Due to financial problems, this center cannot employ nurses for taking care of the elderly. We are inevitable to employ the health care providers who have lower education*” (Participant No. 17).

**Spiritual distress**: Lack of support from family and living in an environment without sufficient support makes the elderly suffer from a range of spiritual distress. Decreased religious commitment, disturbed understanding of meaning and purpose of life, disappointment, decreased internal peace and disturbed communication are the subcategories of this concept.

At this point, as the elderly assume the others guilty for the existing conditions, they experience decreased religious commitment and are no longer committed to praying, saying prayers and their beliefs. For example, one of the elderlies mentioned: “*I said my prayers before, but since I have come here, I feel it is useless. No matter how much I pray, I receive no response and I feel that God doesn't love me anymore*” (Participant No. 13). As the elderly living in nursing homes see themselves discarded by society, they imagine this situation as a type of revenge by destiny and lose their belief in some values. This theme was so evident in the interviews that one of the participants mentioned that: “*I always said to myself that do good to receive good, but now I see that I have made a mistake. I served my parents that much and took care of them, but the result was that my children took me here and left*” (Participant No. 26).

When the old people are transferred to the nursing homes, they assume their peace to be lost. They feel that they have lost the balance they had in their personal life and have no control over their situation. One of the participants mentioned that: “*I am very confused and bored. I had control over everything at my own home, when to sleep, when to awake; but nothing is clear here. Nothing is under your control*” (Participant No. 20).

Also, meaning and purpose of life for some elderly people was unclear and they saw life meaningless and aimless. One of the elderly participants mentioned that: “*I feel my life is very meaningless and aimless here. We only change days with nights, we have no work, no entertainment, nothing*” (Participant No. 29).

Also, some elderly people felt disappointed and had no hope in life. As they mentioned, lack of effective activities and separation from their personal life environment has led to their hopelessness and disappointment. One of the elderly participants mentioned that: “*We have come here to see when death comes to us. Every single day that I awake, I say that today is the last day of my life*” (Participant No. 27).

Also, disturbed communication was another concept formed in this category. Disturbed communication with God, family, other elderly people and health care providers was among the issues evident in the interviews. According to one of the participants, most of his communication with others has been disturbed: “*I have no communication with my family and I have no interest in talking with other elderly people. I don't like to speak with anyone anymore*” (Participant No. 24).

**Seeking support**: In this stage, the elderly seek support following spiritual distress and need for support. Gradually, the elderly pay more attention to spirituality, as if some sort of spiritual awakening occurs in them and start a spiritual journey toward achieving spiritual health. This category is composed of two subcategories of emergence of spiritual needs and reliance on supportive resources.

Emergence of spiritual needs appears in elderly in the form of need for hope, meaning and peace. One of the important questions concerning some elderly people was the reason of their residence in nursing homes. This question can show their spiritual need for understanding the meaning and purpose of life. One of the participants mentioned: “*I don't know why I ended here. I never thought to die in a nursing home. I always thought those who end in nursing homes are very poor and helpless*” (Participant No. 19).

Also, the participants referred to some cases which can indicate their need for hope. For example, one of the participants mentioned: “*I wish someone was here to give us a little hope. When you are new to a nursing home, you need to keep your spirits up*” (Participant No. 8).

Helplessness on one hand and emergence of spiritual needs on the other hand lead to reliance of the elderly on supportive resources, including religious resources and rehabilitation care. These resources are considered as spiritual resources due to their role in creating hope, peace and meaning.

During the interviews, it was revealed that many elderly people use different religious resources, including praying, seeking religious help and belief in God's love. For example, a 69-year woman mentioned that: “*Whenever I feel sad, I talk to God; I pray*” (Participant No. 5).

Also, seeking rehabilitation care such as occupational therapy or psychotherapy was considered as another action taken by the elderly people for using supportive resources. One of the participants mentioned: “I*t has been some days that I practice occupational therapy. I feel better now*” (Participant No. 21).

**Relative improvement of spiritual health**: Reliance on spiritual supportive resources leads to obtaining spiritual pleasure in the elderly, which includes understating the meaning, hope and peace and indicates response to spiritual needs. Also, a kind of spiritual development is evident in the elderly, which can show achieving a relative improvement of spiritual health. Additionally, constructive relationship with others was another concept formed in this category which included communication with God, family, health care providers and other elderly people.

During the interviews, the elderly people referred to multiple factors that provided the context for obtaining spiritual pleasure by them. Some of them were related to religious affairs (such as trust in God's support, belief in miracle and effect of religious vows, praying, intuition and signs and the belief that life and death of human is controlled by God) and some others were related to nonreligious affairs (such as support by family, health care providers and other elderly people). A 72-year old woman defined obtaining spiritual pleasure as this: “*Now my life is better, I feel calm, I'm hopeful*” (Participant No. 4). Also, a 68-year old mentioned: “*It doesn't matter anymore when I am to die, what is important is using the moments of my life*” (Participant No. 16).

Also, it was revealed during the interviews that the existing situation causes some elderly people grow spiritually. In other words, spirituality is reflected in their actions as transcendental attitude (turning to God, appreciating God's gifts more, reduced attention to material things, attention to the afterlife) and avoidance of egocentrism (decision to help, forgive and love other people). A 76-year old woman mentioned that: “*I have forgiven anyone who has done something bad to me. I am not angry with anyone. I hope God forgives me too*” (Participant No. 22).

Also, constructive relationship with others was an important issue to which the elderly referred. One of the participants mentioned: “*I communicate with God more than before. When you feel lonely at an unfamiliar place, you turn to communication with God more*” (Participant No. 5).

**Factors affecting spiritual health**: Factors affecting spiritual health were the factors which influenced the process of spiritual health of the elderly. These factors included personal factors, family factors and care factors.

The type of the spiritual need of the elderly was influenced by their understanding of spirituality, so that there were different spiritual needs depending on the concept of spirituality from their point of views. Most of the elderly in this study believed in the religious dimension of spirituality and used the concepts of communication with God, trust in God and following God's orders and instructions for defining spirituality. To define spirituality, some of the participants referred to ethical dimension as well as religious dimension and considered observing ethical rules in personal and interpersonal contexts as a sign of spirituality in addition to religious practice. An 80-year old man mentioned that: “*As long as you don't observe ethics and don't consider God's satisfaction in your actions, you reach nowhere. In order for your life to be spiritual, you should believe in god and good manner*” (Participant No. 14).

Also, family status of the elderly in terms of affective relationships and their spiritual and religious beliefs were among the factors considered to be influential in spiritual health from the elderly's point of view. One of the elderly mentioned that: “*I was grown up in a religious family. I went to the mosque with my father when I was a child. I participated in spiritual prayers and ceremonies. This caused me to keep my belief in God and spiritual issues even now*” (Participant No. 5).

From the elderly's point of view, care factors were among the items which affect their spiritual health and can be effective in improving or lowering their spiritual health. Recreational camping and pilgrimage, joyous and entertaining programs and holding spiritual ceremonies such as group prayers can lead to improved spiritual health of the elderly. One of the participants mentioned that: “*Last year, we went on a pilgrimage planned by the nursing home. We had so much fun. I experienced a very good spiritual feeling. Even until now, the sweet memories of that trip are with me*” (Participant No. 25).

Altogether, the important concept is the spiritual distress, which shows the need of the elderly for support and can be considered as the central variable. Also, the strategies employed by the elderly (reliance on supportive resources) show their continuous efforts toward obtaining support. Therefore, “effort toward being supported” is considered as this study's theory as a strategy for reducing the consequences of problem.

## Discussion

The analysis of the collected data lead to extraction of 6 main categories, including helplessness, inefficient supportive environment, spiritual distress, seeking support, relative improvement of spiritual health and factors affecting spiritual health. These categories explain the whole process of spiritual health of the elderly living in nursing homes. Helplessness was one of the main categories emerged from the data. From the participants' point of view, lack of family support system, limited income and reduced physical and cognitive abilities were among the factors affecting this concept. In their study, Marsa et al. also found that helplessness in the elderly living in nursing homes was significantly higher than that in other elderly people, which is highly related to their place of residence (20).

Inefficient supportive environment was another category emerged from the data. In such environments, enough spiritual care is not provided and health care providers do not have sufficient knowledge for taking care of the elderly. Ayyari et al. also showed that the elderly living in nursing homes are deprived of essential spiritual care and health care providers are not able to fill the gap resulted from the lack of family support (21). Also, Erichsen et al. believe that spiritual needs of the elderly living in nursing homes are not met sufficiently, a reason of which is assumed to be the insufficient knowledge of health care providers of the elderly's spiritual needs (22).

Spiritual distress was another category emerged from the data. In fact, it can be said that spiritual distress is a consequence of helplessness and being in an inefficient supportive environment, which causes disturbed communication of the individual with self, God and others. It has been indicated that the elderly living in nursing homes, especially at the beginning of their residence in nursing homes, experience some degrees of spiritual distress due to changes made in their life conditions (22–23). Caldeira et al. defined spiritual distress of the elderly as a disturbance in seven structures of the individual's spirituality: connection, religious belief and faith system, value system, meaning and purpose of life, self-transcendence, internal peace and coordination and internal power and energy. They believe that the elderly, compared to the other age groups, are more vulnerable to spiritual distress (24). The study by Li et al. also shows that the elderly living in nursing homes experience spiritual distress more than other elderly people, so that this can affect their other health dimensions adversely (25).

Following helplessness and emergence of spiritual distress in the elderly, they seek the ways to be able to get out of this situation. Therefore, the strategy employed by them is seeking support. At this stage, the elderly search for supportive resources to rely on (including religious resources and rehabilitation care) and express their spiritual needs, including need for meaning, hope and peace. Hence, they achieve some degrees of spiritual awakening. The Noble Quran has also mentioned that whenever the humans are suffering by pain and hardship, they call their God. This finding is consistent with the results of a study which indicated that following the spiritual lacks, resulted from the residence in nursing homes, the elderly seek spiritual support and try to meet their spiritual needs through different ways (26). Over time and in the event that their search for support is successful, the elderly's spiritual health relatively improves. Consequently, they understand spiritual pleasures, turn to constructive relationships with others and their spiritual distress is reduced, and the result of these changes is adaptation to living in nursing home. Of course, some elderly people reach spiritual growth and reach a higher level of spiritual health (27). Matsubayashi et al. also believe that passage of time can lead to improved adaptation of the elderly to living in nursing homes and promote their spiritual health (28). However, some studies show that the elderly living in nursing homes for a long period of time express a lower level of spiritual health (14, 29). This inconsistency in results can be due to differences in the participants' age and their different health levels.

Another important category emerged in this study was the factors affecting spiritual health of the elderly, which compasses a wide range of factors. These factors are divided into three personal, family and care subcategories, each of which can be influential in improving the level of spiritual health of the elderly. Spiritual background of the elderly individual and his family as well as provision of spiritual care by nurse and health care providers are among the factors improving the spiritual health and lack of belief in religion by the individual, having a disorganized family and lack of spiritual care in nursing home are among the factors which inhibit the improvement of the elderly's spiritual health. In this regard, Tsuboi et al. also believe that spiritual health of the elderly is, to a large extent, a result of their spiritual performance in their previous years of life, so that commitment of the elderly people since their young to middle age determines the extent of their spirituality in old age (30). Also, Touhy et al. indicate that although the background of the elderly individual can determine his level of spiritual health, provision of spiritual care to these people can lead to their enhanced spiritual health. They emphasize that health care providers taking care of the elderly should consider the factors affecting their spiritual health (31).

The analysis of the collected data led to extraction of six categories explaining the process of spiritual health of elderly lining in nursing homes. These categories included helplessness, inefficient supportive environment, spiritual distress, seeking support, relative improvement of spiritual health and factors affecting spiritual health. Explaining the process of spiritual health in practice can lead to provision of guidance for nurses, physicians and other health care providers in order to provide more appropriate care to the elderly living in nursing homes.
